# Air-Liquid-Interface Differentiated Human Nose Epithelium: A Robust Primary Tissue Culture Model of SARS-CoV-2 Infection

**DOI:** 10.3390/ijms23020835

**Published:** 2022-01-13

**Authors:** Bang M. Tran, Samantha L. Grimley, Julie L. McAuley, Abderrahman Hachani, Linda Earnest, Sharon L. Wong, Leon Caly, Julian Druce, Damian F. J. Purcell, David C. Jackson, Mike Catton, Cameron J. Nowell, Laura Leonie, Georgia Deliyannis, Shafagh A. Waters, Joseph Torresi, Elizabeth Vincan

**Affiliations:** 1 Department of Infectious Diseases, Melbourne Medical School, Faculty of Medicine, Dentistry and Health Sciences, The University of Melbourne at the Peter Doherty Institute for Infection and Immunity, Melbourne, VIC 3000, Australia; manht@unimelb.edu.au; 2 Department of Microbiology and Immunology, School of Biomedical Sciences, Faculty of Medicine, Dentistry and Health Sciences, The University of Melbourne at the Peter Doherty Institute for Infection and Immunity, Melbourne, VIC 3000, Australia; samantha.grimley@unimelb.edu.au (S.L.G.); jmcauley@unimelb.edu.au (J.L.M.); abderrahman.hachani@unimelb.edu.au (A.H.); linda.earnest@unimelb.edu.au (L.E.); dfjp@unimelb.edu.au (D.F.J.P.); davidcj@unimelb.edu.au (D.C.J.); georgia.deliyannis@unimelb.edu.au (G.D.); 3 Molecular and Integrative Cystic Fibrosis Research Centre, School of Women’s and Children’s Health, Faculty of Medicine, University of New South Wales, Sydney, NSW 2052, Australia; sharon.l.wong@unsw.edu.au (S.L.W.); shafagh.waters@unsw.edu.au (S.A.W.); 4 Victorian Infectious Diseases Reference Laboratory at the Peter Doherty Institute for Infection and Immunity, Melbourne, VIC 3000, Australia; leon.caly@vidrl.org.au (L.C.); julian.druce@mh.org.au (J.D.); mike.catton@mh.org.au (M.C.); 5 Imaging, FACS and Analysis Core, Monash Institute of Pharmaceutical Sciences, Faculty of Pharmacy and Pharmaceutical Sciences, Monash University, Parkville, VIC 3052, Australia; cameron.nowell@monash.edu; 6 Melbourne Histology Platform, School of Biomedical Sciences, Faculty of Medicine, Dentistry and Health Sciences, The University of Melbourne, Parkville, VIC 3010, Australia; lleone@unimelb.edu.au; 7 School of Women’s and Children’s Health, Faculty of Medicine and Health, University of New South Wales, Sydney, NSW 2052, Australia; 8 Department of Respiratory Medicine, Sydney Children’s Hospital, Randwick, NSW 2031, Australia; 9 Curtin Medical School, Curtin University, Perth, WA 6102, Australia

**Keywords:** SARS-CoV-2, COVID-19, human nasal epithelium, air–liquid-interface, organoids

## Abstract

The global urgency to uncover medical countermeasures to combat the COVID-19 pandemic caused by the severe acute respiratory syndrome-coronavirus 2 (SARS-CoV-2) has revealed an unmet need for robust tissue culture models that faithfully recapitulate key features of human tissues and disease. Infection of the nose is considered the dominant initial site for SARS-CoV-2 infection and models that replicate this entry portal offer the greatest potential for examining and demonstrating the effectiveness of countermeasures designed to prevent or manage this highly communicable disease. Here, we test an air–liquid-interface (ALI) differentiated human nasal epithelium (HNE) culture system as a model of authentic SARS-CoV-2 infection. Progenitor cells (basal cells) were isolated from nasal turbinate brushings, expanded under conditionally reprogrammed cell (CRC) culture conditions and differentiated at ALI. Differentiated cells were inoculated with different SARS-CoV-2 clinical isolates. Infectious virus release into apical washes was determined by TCID_50_, while infected cells were visualized by immunofluorescence and confocal microscopy. We demonstrate robust, reproducible SARS-CoV-2 infection of ALI-HNE established from different donors. Viral entry and release occurred from the apical surface, and infection was primarily observed in ciliated cells. In contrast to the ancestral clinical isolate, the Delta variant caused considerable cell damage. Successful establishment of ALI-HNE is donor dependent. ALI-HNE recapitulate key features of human SARS-CoV-2 infection of the nose and can serve as a pre-clinical model without the need for invasive collection of human respiratory tissue samples.

## 1. Introduction

Over the past 15 years, 90% of novel medical countermeasures that showed promise in preclinical animal and cell line models failed in human clinical trials: 50% for lack of efficacy, 30% for toxicity [1,2]. Importantly, the toxicity was not detected in non-human primates, the closest animal model to humans. This failure rate continues to this day. To improve our understanding of host–pathogen interactions, we need to advance our pre-clinical models to better reflect human physiology.

Human adult stem cell-derived organoids fill the gap between animal and cell line pre-clinical models, and human clinical trials. Tissue stem cells ‘remember’ their tissue of origin, they generate the same cell types in a dish as they do in the body and recapitulate key features of architecture and function of the parent tissue [3,4,5,6,7,8]. Early studies by the Clevers [9] and Estes [10] laboratories exemplified the power of tissue stem cell derived organoids for modelling lung (respiratory syncytial virus (RSV)) and gut (human noroviruses (HuNoVs)) infection, respectively. In a similar vein, induced pluripotent stem (iPS) cell derived organoids proved invaluable for understanding the pathogenesis of Zika virus (ZIKV) in the brain [11,12]. Unlike respiratory and gastrointestinal tissues, which can be sourced from resected tissues from routine surgical procedures and non-invasive sample collection (e.g., nasal turbinate brush), human brain tissue is much harder to source, highlighting the importance of iPS technology for less accessible tissues. However, the advantages that these human organoids provide in modelling human viral infectious diseases remained largely overlooked in preference for decades old virus culture systems.

Early during the COVID-19 pandemic when medical countermeasures were being assessed, virologists used classical cell lines such as Vero cells for neutralisation and anti-viral studies. The Vero cell line was derived from African Green Monkey kidney epithelial cells [13] and has been the workhorse of virology laboratories since the 1960s. Vero cells contain genomic deletions of genes involved in the antiviral interferon response [14] and are thus highly susceptible to infection by diverse viruses, including SARS-CoV-2, yielding high virus titres. However, SARS-CoV-2 viral entry into Vero cells differs markedly from entry into human epithelial cells [15]. The SARS-CoV-2 spike glycoprotein mediates viral entry into Vero and primary epithelial cells by binding to the human angiotensin-converting enzyme-2 (ACE-2) [16,17]. However, entry into primary epithelial cells requires proteolytic cleavage by the cellular protease TMPRSS2 which initiates direct fusion between cellular and viral membranes, whereas entry into Vero cells is via an endosomal pathway [15]. Thus, drugs like hydroxychloroquine, which inhibits endosomal acidification, showed robust efficacy in Vero cells [18,19] but lacked efficacy in human clinical trials [20]. Had the original studies been performed in human epithelial organoids, hydroxychloroquine would not have been considered a viable candidate for further clinical trials. Furthermore, primary human cell responses to SARS-CoV-2 infection are not recapitulated in animal models, human continuous cell lines, or Vero cells [21,22]. Consequently, results with antivirals and neutralising antibodies obtained in these models fail to reflect responses in humans. Additionally, deep sequencing of SARS-CoV-2 isolates and culture supernatant preparations demonstrated that propagation in cell lines, such as Vero cells, led to mutation of the viral genome adapting the virus for growth in simple tissue culture cells [23,24]. Again, this confounds the validity of pre-clinical assays performed with such culture systems that a far removed from human tissue.

Unsurprisingly, the global urgency to develop vaccines and antivirals to combat COVID-19 has exposed the shortcomings of decades old tissue culture methods used by virologists and has seen an exponential increase in the adoption of organoids from many human tissues to understand pathogenesis and test therapies [25]. The first report to demonstrate that human adult stem cell (hASC) derived organoids are productively infected by SARS-CoV-2 was a collaboration between the Clevers and Haagmans laboratories in the Netherlands, who showed robust infection of primary gut and respiratory epithelium [26]. Within a year of this publication, Chen and colleagues reported on human pluripotent stem cell (hPSC) derived alveoli-like and colon epithelium-like organoid-based screens for SARS-CoV-2 inhibitors [27]. hPSC derived organoids have the advantage of being a renewable source, but they do not recapitulate human tissue architecture to the degree achieved by tissue stem/progenitor cell derived organoids [9,28,29].

To circumvent these caveats, we revisit a well-established and characterized upper respiratory epithelium model [30,31,32]. The human nasal epithelium (HNE) is considered the first site of SARS-CoV-2 infection and expresses high levels of ACE2 [33], the cell surface receptor for SARS-CoV-2 [16,17], and is thus potentially the ideal tissue for testing prevention of virus entry into the body. To this end, we have established and characterized an air–liquid-interface (ALI) differentiated HNE model for SARS-CoV-2 infection. We demonstrated robust SARS-CoV-2 infection of ALI-HNE established from low passage progenitors from most donors (6/9) and observed increased cell damage by the Delta variant clinical isolate compared to an ancestral clinical isolate from January 2020 [34]. Unlike other human tissues, the non-invasive sample required (nasal turbinate brush) and commercially available media makes ALI-HNE an attractive model system for respiratory viruses.

## 2. Results

### 2.1. SARS-CoV-2 Infects ALI-HNE Established from Adult and Child Donors

To evaluate ALI-HNE culture as a model for SARS-CoV-2 infection, turbinate brush samples were collected from adult and child donors and infected with SARS-CoV-2 clinical isolates (Table 1). ALI-HNE established from conditionally reprogrammed epithelial cells generated from the nasal turbinate brush samples from an adult (PDI-1) and child (PDI-7) donor yielded pseudostratified epithelium several layers deep. As previously described, the well-developed apical cilia were detected by staining for acetylated α-tubulin (AcTub) [35]. The orthogonal view of the immunofluorescent confocal microscopy Z-sections, and the movie generated from the Z-sections (Videos S1 and S2), clearly demonstrated deep layering with apical cilia (Figure S1). Light microscopy live cell time-lapse imaging revealed beating cilia (Videos S3 and S4).

**Figure 1:**
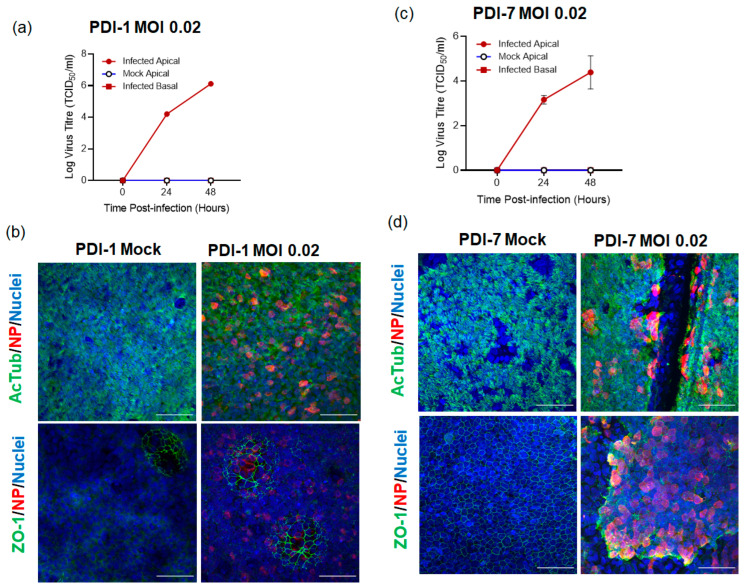
SARS-CoV-2 infected ALI-HNE. Adult (**a**,**b**) and child (**c**,**d**) infected with VIC01. (**a**,**c**) Infectious virus (TCID_50_) in the apical wash and basal medium harvested at the indicated times; (**b**,**d**) Immunofluorescent confocal microscopy staining for α-tubulin (AcTub, green) and nucleoprotein (NP, red). Nuclei are blue (DAPI). Scale bar 50 µm.

**Figure 2:**
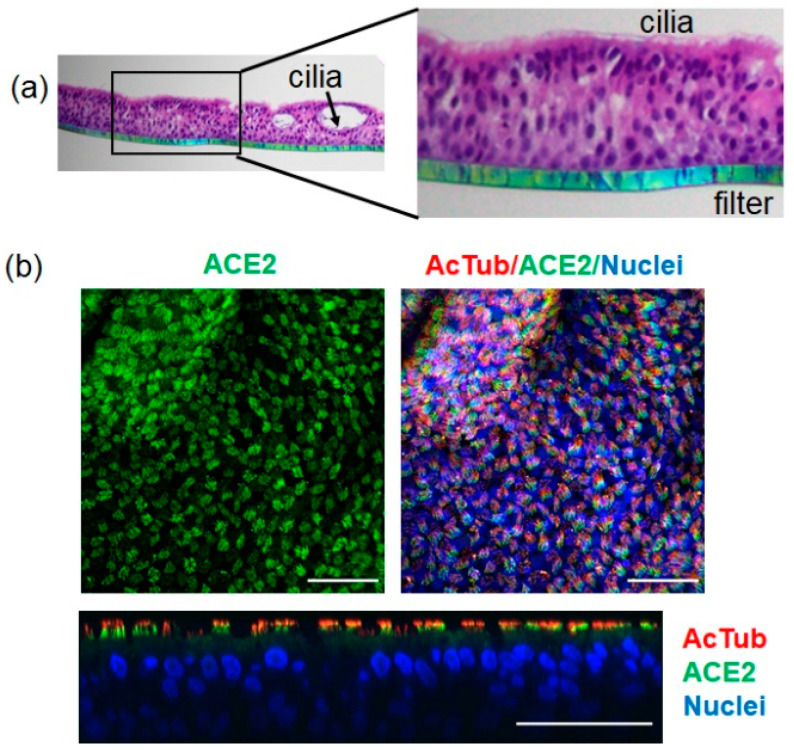
ACE2 is expressed by ALI-HNE. (**a**) Hematoxylin and eosin staining shows apical cilia and deep pseudostratified differentiation. (**b**) Immunofluorescent staining for ACE2 (green) and α-tubulin (red); nuclei are blue (DAPI). Top and orthogonal views are shown. Scale bar 50 µm.

Both adult (Figure 1a,b) and child (Figure 1c,d) ALI-HNE cultures were susceptible to infection by the Australian ancestral SARS-CoV-2 clinical isolate, VIC01 [34], at an MOI of 0.02. Virus infected cells were detected by staining for viral nucleoprotein by immunofluorescent confocal microscopy (Figure 1). Extended data for the immunofluorescent confocal microscopy and staining with control antibodies is shown in Figure S2. Infectious virus, quantified by TCID_50_ on Vero cells, was detected in the apical wash harvested at the indicated times but not in the basal medium (Figure 1a,c). Staining for ZO-1 revealed well-developed tight junctions (Figure 1b,d). Interestingly, spheroid organoids were formed within the pseudostratified epithelium (shown by ZO-1 staining of PDI-1, Figure 1b, and Videos S3 and S4). The extent of spheroid formation within the ALI-HNE was donor dependent where only a few were seen in PDI-1 (Figure 1b), for example, and many in PDI-4 (Figure 2a).

### 2.2. SARS-CoV-2 Infection of ALI-HNE Is Dose Dependent

To further characterise the infectivity of SARS-CoV-2 clinical isolates on ALI-HNE, we focused on adults as this is a less limited resource than child donors (Table 1). Haematoxylin and eosin staining of formalin fixed paraffin embedded PDI-4 filters confirmed deep pseudostratified differentiation with apical cilia and the formation of spheroids within the epithelium. Cilia were on the luminal side of the spheroids (Figure 2a). ACE2 was expressed at the apical surface of the ALI-HNE (Figure 2b), consistent with the expression pattern in primary intestinal epithelium [26]. The orthogonal view of the confocal Z-sections placed ACE2 at the base of the cilia. Extended data for the immunofluorescent confocal microscopy and staining with control antibodies is shown in Figure S3.

Notably, when PDI-4 and PDI-5 basal cells were embedded in BME2 matrix for spheroid organoid culture, and expanded and differentiated in PneumoCult^TM^ Organoid media, differentiated cells formed spheroids with apical surface towards the matrix (PDI-4, Video S5). The spheroids with cilia on the outside of the organoid would sometimes spin (PDI-5, Video S6). Apical-out organoids provide an alternative HNE model as these types of cultures can be generated to be ever-expanding and cryopreserved at the expansion phase using previously described protocols [9].

**Figure 3:**
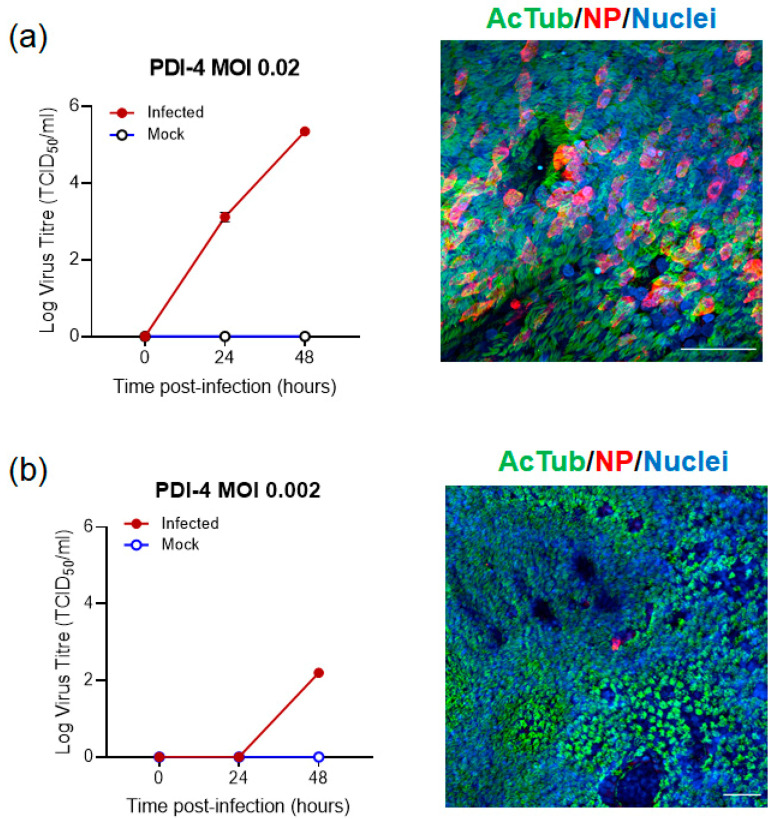
Dose dependent SARS-CoV-2 infection of ALI-HNE. ALI-HNE infected with VIC01 at MOI 0.02 (**a**) and 0.002 (**b**). Infectious virus (TCID_50_) in the apical wash harvested at the indicated times; and immunofluorescent confocal microscopy staining for α-tubulin (AcTub, green) and nucleoprotein (NP, red). Nuclei are blue (DAPI). Scale bar 50 µm.

Next, we tested the dose dependence of infection by infecting PDI-4 (Figure 3 and Figure S4) and PDI-2 (Figure S5) ALI-HNE with VIC01 at an MOI of 0.02 and 0.002. Infectious virus was detected at the indicated times in the apical washes by TCID_50_ on Vero cells (Figure 3 and Figure S5a,d), while infected cells were visualised by staining for nucleoprotein (Figure 3 and Figure S5b,e). Infectious virus was detected 24 h post-infection at an MOI of 0.02 but not MOI of 0.002 for both PDI-4 and PDI-2 ALI-HNE. Extended data for the immunofluorescent confocal microscopy and staining with control antibodies is shown in Figure S4 for PDI-4. The orthogonal view of the confocal Z-sections shows nucleoprotein was primarily apical at 48 h post infection (Figure S5b,e).

Next, we tested ALI-HNE susceptibility to infection when passaged in T25 flasks to expand the basal cells. Passaged PDI-1 cells showed delayed virus production despite differentiating well at ALI, while PDI-2 failed to differentiate at ALI after passage (Table 1). PDI-6 failed to differentiate at ALI on several attempts, while PDI-3 cultures had mixed cells type (ciliated and non-ciliated cells) and infected poorly or not at all with VIC01 (Figure S6). Collectively, these data demonstrate that achieving good differentiation at ALI for robust SARS-CoV-2 infection requires low passage CRC basal cells.

### 2.3. The SARS-CoV-2 Delta Variant Infects ALI-HNE

**Figure 4:**
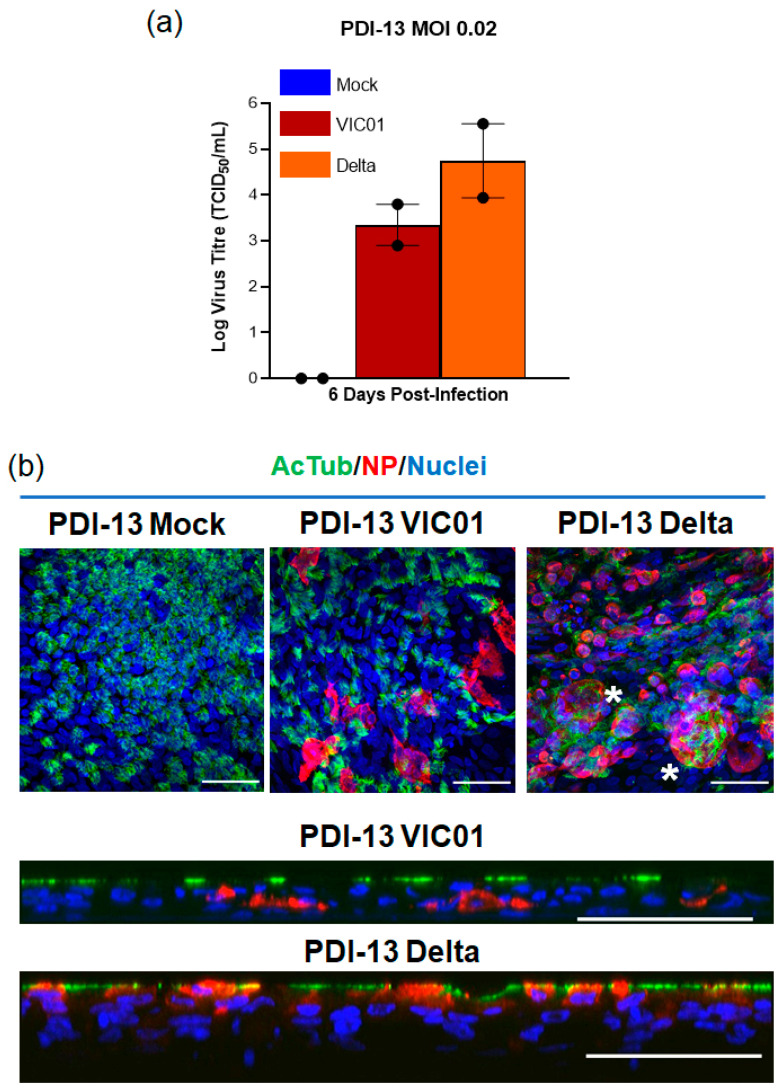
VIC01 and Delta SARS-CoV-2 infected ALI-HNE. (**a**) Infectious virus (TCID_50_) in the apical wash harvested 6 days post infection; (**b**) Immunofluorescent confocal microscopy staining for α-tubulin (AcTub, green) and nucleoprotein (NP, red). Nuclei are blue (DAPI). * Indicates examples of syncytia formation. Top and orthogonal views are shown. Scale bar 50 µm.

The nasal epithelium is considered the first site of infection during SARS-CoV-2 pathogenesis, thus assessing infection of ALI-HNE cells by variants of SARS-CoV-2 as they emerge might provide insight on the transmissibility of mutant viruses, termed variants of concern (VOC). During the pandemic, we have tested the susceptibility of ALI-HNE to mutant SARS-CoV-2 variants (D614G, Alpha, and Beta) and demonstrated that each variation from the ancestral strain infects ALI-HNE (data not shown). Here, we focus on the Delta variant; it first emerged in India late 2020 and has swept across the globe, rapidly outcompeting the pre-existing lineages deemed VOC [36,37]. The Delta spike was shown to facilitate more cell–cell fusion kinetics and syncytia formation compared to Wuhan-1 [38,39]. Here we compared Delta and VIC01 infection of ALI-HNE at an MOI of 0.02. Infectious virus was not detected by TCID_50_ assay at 24 h or 48 h post-infection with either virus with this donor (PDI-13); however, productive infection was observed at 6 days with both SARS-CoV-2 lineages. Delta infected cultures produced approximately 10-fold more virus than the VIC01 infected cultures (TCID_50_, Figure 4a). Delta infection was more cytopathic with syncytia and extensive nuclear damage (Figure 4b). Extended data for the immunofluorescent confocal microscopy and staining with control antibodies is shown in Figure S7. These data are consistent with recent observations with Delta infection of HNE cells and hamsters [38,39].

## 3. Discussion

The failure to translate from a pre-clinical model to human clinical trial raises the cost per new drug to $US2.8 billion [1,2]. Furthermore, it means patients and clinical trial volunteers are treated with drugs that will not work in humans to prevent or treat disease. Alarmingly, toxicity in humans can be fatal as was seen with an HBV antiviral (fialuridine, FIAU) due to toxicity to a human mitochondrial gene, which was not seen in pre-clinical animal models, including non-human primates [40,41]. The promise of human tissue stem cell-derived organoids is to fill the gap between animal and cell line pre-clinical models and humans [3,4,5,8]. Organoids recapitulate key features of human tissue in a dish and thus offer physiologically relevant models of host–virus interaction. Indeed, the unprecedented global rush to develop medical countermeasures to combat the COVID-19 pandemic has revealed the shortcomings of classical tissue culture models used by virologists. Hydroxychloroquine showed promise as an antiviral in Vero cells, but did not prevent infection of primary epithelial cells and failed human clinical trial [18,19,20].

SARS-CoV-2 is thought to enter the body mainly via the upper respiratory tract, however, COVID-19 is a systemic disease affecting the gut, liver, heart, brain, endovascular system, etc. and organoids from diverse tissues and organs have been used to understand SARS-CoV-2 pathogenesis (reviewed in [42,43,44]). Given that current vaccines work systemically, SARS-CoV-2 must infect the body for the vaccines to reduce COVID-19 morbidity and mortality [45]. We anticipate that the goal of second and third generation medical countermeasures will be sterilizing vaccination and treatments against SARS-CoV-2 that prevent or control infection of the HNE, the main portal of entry into the body. To this end, we have shown that ALI-differentiated HNE were susceptible to infection by SARS-CoV-2 and recapitulated key features of human infection. The ALI-HNE express ACE2 on their apical surface; viral entry and release were via the apical surface. This is consistent with previous observations, demonstrating ACE2 and TMPRSS2 expression in human nasal epithelium [33,46]. Furthermore, we showed ALI-HNE established from adult and child donors were susceptible to infection. SARS-CoV-2 infection was dose dependent with faster viral production at an MOI of 0.02 than 0.002. Virus production was also donor dependent and required low passage nasal progenitor cells. Infection by the Australian ancestral strain, VIC01 [34], did not lead to overt cytopathic effect. In stark contrast, infection of the same ALI-HNE with the Delta variant led to extensive syncytial formation. Enhanced fusogenicity of the SARS-CoV-2 Delta variant that we observed is consistent with recent reports [38,39]. The enhanced pathogenicity phenotype of the Delta variant revealed by the ALI-HNE might underlie the increased transmissivity of this variant in human populations and its global predominance [36,37]. 

The Omicron SARS-CoV-2 variant emerged in South Africa in late 2021 [47] and is now rapidly spreading globally. Omicron has >30 mutations in the spike protein. Infection studies in lung organoids show compromised replication and pathogenicity [48]. The pathogenicity phenotype of the Omicron variant in ALI-HNE and donor-to-donor variation remains to be fully characterized, but a recent study using a commercial source of ALI-HNE shows rapid viral kinetics and the potential for TMPRSS2-independent entry [49]. The latter might underlie Omicron’s enhanced intrinsic transmissibility via the upper respiratory tract, while the compromised replication and pathogenicity in the lower respiratory tract might underlie the decreased mortality and morbidity [47,50].

Collectively, these data show the ALI-HNE is a faithful model of SARS-CoV-2 infection and can predict pathogenicity of mutant SARS-CoV-2 variants. The non-invasive sample required and the relatively straightforward culture conditions (i.e., standard humidified CO_2_ incubators and commercially available media) mean that variants can be screened in real-time as they emerge once a Biobank of basal progenitors is established. Our study shows that donors need to be pre-screened to ensure that their nasal turbinate brush samples yield well-differentiated ALI-HNE and support robust, productive SARS-CoV-2 infection. Adoption of this culture system into a 96-well format will facilitate high throughput screens for medical countermeasures. Furthermore, in future studies, ALI-HNE from different donors coupled with omics analyses might reveal the molecular mechanisms underlying donor variation in drug responses and antibody neutralization. Organoid platforms like the ALI-HNE make personalized COVID-19 countermeasures a reality. Finally, the rapid adoption of organoids established from diverse human tissues is likely to not only reveal novel avenues to prevent or control systemic COVID-19, but also shed light on alternative routes of entry into the body. The occurrence of SARS-CoV-2 infection, despite wearing a face mask to protect the respiratory route of entry, raises the possibility that the virus can enter the body via an alternative route such as the eye [51,52,53]. The human ocular tissue expresses ACE2 and TMPRSS2 [54,55] and SARS-CoV-2 infection of human ocular tissue has been demonstrated [55]. Consequently, these studies in diverse human organoid models guide public health measures—e.g., personal protective equipment—as well as rigorously test the efficacy of COVID-19 medical countermeasures.

## 4. Materials and Methods

### 4.1. Procurement of Human Material and Informed Consent

Study approval was received from the Sydney Children’s Hospital Network Ethics Review Board (HREC/16/SCHN/120) and the Medicine and Dentistry Human Ethics Sub-Committee, University of Melbourne (HREC/2057111). Written consent was obtained from all participants (or participant’s guardian) prior to collection of biospecimens.

### 4.2. Primary Nasal Epithelium Culture and Differentiation

De-identified, cryopreserved human nasal epithelial cells were received from the Molecular and Integrative Cystic Research Centre (miCF RC), University of New South Wales, New South Wales, Australia where they were harvested from nasal turbinate brush samples with donor consent and cultured under conditional reprogram conditions (CRC) as previously described [30,31,35,56].

To initiate mucociliary differentiation at the air–liquid interface (ALI), cryovials of cells (500,000 cells/vial) were thawed and seeded onto Transwell inserts (6.5 mm Corning, Kennebunk, ME, USA; three inserts per vial) pre-coated with collagen type I (PureCol-S, Advanced BioMatrix, San Diego, CA, USA). Cells were incubated submerged in PheumoCult^TM^-ExPlus (STEMCELL Technologies, Vancouver, BC, Canada) until confluent, typically 4–7 days, then switched to ALI conditions by removing apical media and adding PneumoCult^TM^ ALI medium (STEMCELL Technologies) to the basal chamber. The basal medium was replaced 3 times per week for 3–4 weeks during which time beating cilia and mucous production were monitored by light microscopy.

To establish matrix-embedded organoids, cryovials of cells (500,000 cells/vial) were thawed and resuspended in 500 µL BME2 (Cultrex Reduced Growth Factor Basement Membrane Matrix, R&D Systems, Minneapolis, MN, USA) on ice and 50 µL domes added per well of 24 well plates. Plates were incubated at 37 °C for 20 min to allow the BME2 to set, then 500 µL of PneumoCult^TM^ Airway Organoid Seeding Medium (AOSM, STEMCELL Technologies) was added per well to cover the domes. AOSM was changed every other day for 7 days. After 7 days, the medium was replaced with PneumoCult^TM^ Airway Organoid Differentiation Medium (AODM, STEMCELL Technologies) to initiate mucociliary differentiation over 3–4 weeks. AODM medium was replaced 3 times a week. Beating cilia and mucous production were monitored by light microscopy.

To expand the basal progenitors, cryovials of cells (500,000 cells/vial) were thawed and seeded onto tissue culture flasks (T-25, Greiner; 1 vial/flask) pre-coated with collagen I (PureCol S). The cells were maintained in PheumoCult^TM^-ExPlus (STEMCELL Technologies) until 80–90% confluent with medium change every other day. Cells were detached with TripLE^TM^ Express Enzyme (Gibco, Life Technologies, UK), seeded into Transwells as above (150,000 cells/filter), and differentiated at ALI as above.

### 4.3. Live Cell Imaging

Images were captured on a Nikon TiE microscope running Nikon NIS Elements Version 5.2 using a 40× PlanApo NA0.75 objective. A CoolSNAP Myo CCD camera set to 4 × 4 binning to achieve 10 frames per second capture was used to generate 10 s movies of cilia beating in the cultures (movie 3 and 4). Alternatively, images were captured on an Olympus CKX41 microscope running CellSens software using SC30 camera.

### 4.4. SARS-CoV-2 Propagation and ALI-HNE Infection

Human SARS-CoV-2 clinical isolates BetaCoV/Australia/VIC01/2020 [34] (referred to as VIC01)) and BetaCoV/Australia/VIC18440/2021 (referred to as Delta) were propagated on Vero cells (ATCC) in DMEM (Gibco), supplemented with 1 μg/mL TPCK-Trypsin (Trypsin-Worthington), HEPES, Glutamax, penicillin (100 IU/mL), and streptomycin (100 IU/mL) at 37 °C in a humidified CO_2_ incubator. Vero cells were seeded in cell culture flasks and infected at a multiplicity of infection (MOI) of 0.01. Supernatant was harvested 72 h later and clarified by low-speed centrifugation. Viral inoculum was aliquoted and stored at −80 °C until use. Infectious virus titers in stocks and samples were determined using TCID_50_; briefly, 10-fold serial dilutions were added to 2 × 10^4^ Vero cells seeded 24 h prior in a 96-well plate. Plates were incubated at 37 °C in a humidified CO_2_ incubator for 3 days and then examined for cytopathic effect (CPE). The TCID_50_ was calculated according to the method of Reed and Muench [57]. Work with infectious SARS-CoV-2 virus was performed in a Class II Biosafety Cabinet under BSL-3 containment at the Doherty Institute for Infection and Immunity.

To infect ALI-HNE with SARS-CoV-2, virus was added to the apical surface at an MOI of 0.02 or 0.002 in 30 µL of inoculum per insert (assuming ~300,000 cell at the ALI-HNE surface [35]). After virus adsorption for 2 h at 37 °C, the inoculum was washed off with PBS containing calcium and magnesium (PBS^++^). Two hundred microliters of PBS^++^ was then added to the apical surface and harvested after 10 min at 37 °C, before being stored at −80 °C. Apical PBS^++^ washes were harvested in the same way at the indicated time points. Apical PBS^++^ wash samples and basal medium collected at time of medium change were assayed for infectious virus by TCID_50_ as above.

### 4.5. Immunofluorescence and Confocal Microscopy

At the indicated experimental endpoints, the cells were washed thrice with PBS^++^ at room temperature. Cells were fixed with 4% paraformaldehyde (Electron Microscopy Sciences, Hatfield, PA, USA) for 30 min at room temperature. The fixative was aspirated and neutralized with 100 mM glycine in PBS^++^ for 10 min at room temperature. Cells were incubated with permeabilization buffer (PB, 0.5% Triton-X in PBS^++^) for 30 min on ice. The PB was washed off with 3 washes of PBS^++^, 5 min each. At this stage, the filters were excised from the inserts using a sharp scalpel, cut in half (for test and control primary antibodies), and transferred to Eppendorf tubes and incubated for 90 min at 4 °C in immunofluorescence buffer (IF, PBS^++^ with 0.1% bovine serum albumin, 0.2% Triton, 0.05% Tween 20) containing 10% normal goat serum (BB, block buffer). At the end of incubation, the BB was removed, and primary antibody diluted in BB added. Following 48 h incubation at 4 °C, the primary antibody was washed off with IF buffer, three times, 5 min each. Fluorophore conjugated secondary antibody and Hoechst, diluted in BB, were added and tubes incubated for 3 h at room temperature. List of antibodies used in is Table S1. Secondary antibody was washed off with IF buffer, five times, 5 min each. Filters were transferred to slides, incubated for 30 min at room temperature with DAPI, washed once with PBS, and mounted in FluoroSave Reagent (EMD Millipore, Billerica, MA, USA); coverslips were sealed with nail polish. The confocal microscopy imaging was acquired on the Zeiss LSM 780 system. The acquired Z-sections were stacked and processed using ImageJ software. Orthagonal views were generated in ZEN 3.1 Software by ZEISS Microscopy.

### 4.6. Immunohistochemistry

The Transwell inserts were washed in PBS^++^, 3 times, 5 min each and the well and insert flooded with 10% neutral buffered formalin (Australian Biostain, Traralgon, VIC, Australia). The fixative was rinsed off with PBS, 3 times, 5 min each. The inserts were dehydrated through an ethanol graded series (35%, 50%, 70%, 95%, 100%, 100%) 10 min each. This was followed by histolene for 10 min, then liquid paraffin (58 °C) was added to the wells and Transwell inserts, and incubated for 1 h, after which time the paraffin was replaced and the 1 h incubation repeated. The plates were removed from the incubator to allow the paraffin to solidify; the membrane with paraffin attached was excised from the insert and embedded into paraffin block and processed using standard histological procedure. Sections (5 µm) were cut and hematoxylin and eosin stain performed using a standard histological protocol.

## Figures and Tables

**Table 1:** SARS-CoV-2 infection of ALI-HNE.

Donor	Sex/Age (yr)	Virus ID	MOI	TCID50 (log 10)	ALI Differentiation
PDI-1	F/32	VIC01	0.02	24 h: 4.2/4.2 48 h: 6.0/6.2	Good
PDI-1 P2 ^	F/32	VIC01	0.02	24 h: LOD */LOD 144 h: 2.94/3.6	Good
			0.02	24 h: LOD/LOD 144 h: 4.94/3.27	
PDI-3	M/44	VIC01	0.02	24 h: LOD/LOD/LOD 48 h: 3.44/3.27/3.6	Medium
			0.02	24 h: LOD/LOD/LOD 48 h: LOD/LOD/LOD	
PDI-4	M/26	VIC01	0.02	24 h: 3.0/3.2 48 h: 5.3/5.4	Good
			0.002	24 h: LOD/LOD 48 h: 3.44/3.27	
PDI-2	M/56	VIC01	0.02	24 h: 3.6/3.1 48 h: 4.3/4.6	Good
			0.002	24 h: LOD/LOD 48 h: 3.1/2.2	
PDI-2 P2	M/56	N/A #	N/A	N/A	Failed to ALI differentiate
PDI-7	F/13	VIC01	0.02	24 h: 3.3/2.9 48 h: 4.1/3.8	Good
PDI-6	F/57	N/A	N/A	N/A	Failed to ALI differentiate
PDI-13	M/37	VIC01	0.02	24 h: LOD/LOD 48 h: 2.6/LOD 144 h: 3.8/2.9	Good
		VIC18440 (Delta)	0.02	24 h: LOD/LOD 48 h: 2.7/1.0 144 h: 3.9/5.6	

NOTE: ^ Passage number; * Limit of Detection (LOD); # not applicable (N/A).

## Data Availability

Not applicable.

## Supplementary Materials

The following supporting information can be downloaded at: https://www.mdpi.com/article/10.3390/ijms23020835/s1.
