# Supplementary material for: Air-Liquid-Interface Differentiated Human Nose Epithelium: A Robust Primary Tissue Culture Model of SARS-CoV-2 Infection
Source: Int J Mol Sci. 2022 Jan 13;23(2):835. doi: 10.3390/ijms23020835 (PMC8776210; doi:10.3390/ijms23020835)
Supplement: Supplementary file 1 [file ijms-23-00835-s001.zip › supplementary/ijms-1544456-supplementary.pdf]

International Journal of
Molecular Sciences

Supplementary Materials: The following are available online at www.mdpi.com/xxx/s1,

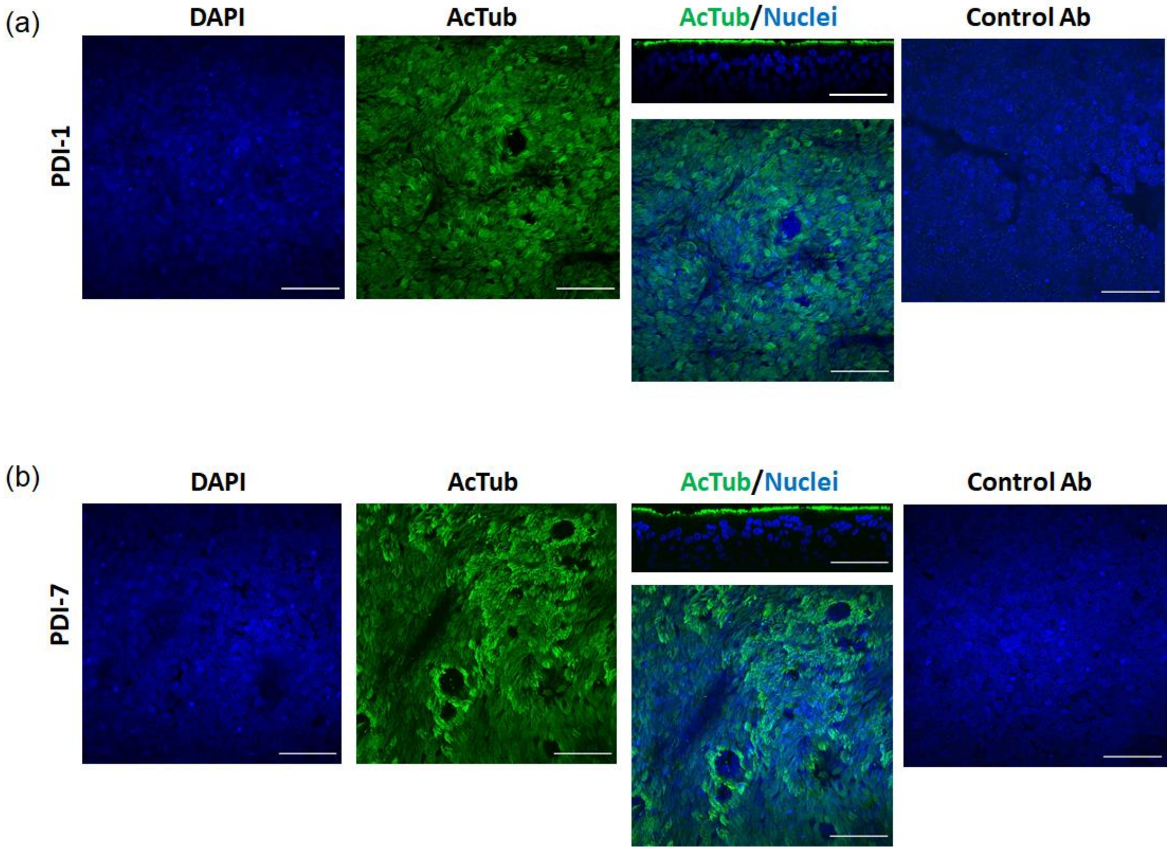

Figure S1. Human nasal epithelium differentiation at air-liquid interface. Adult (**a**) and child (**b**) air-liquid interface (ALI) differentiated human nasal epithelium (HNE) was characterized by immunofluorescent confocal microscopy staining for α-tubulin (AcTub, green) and DAPI for nuclei (blue) or control antibody (Control Ab). Top and orthogonal views are shown. Scale bar 50 µm.

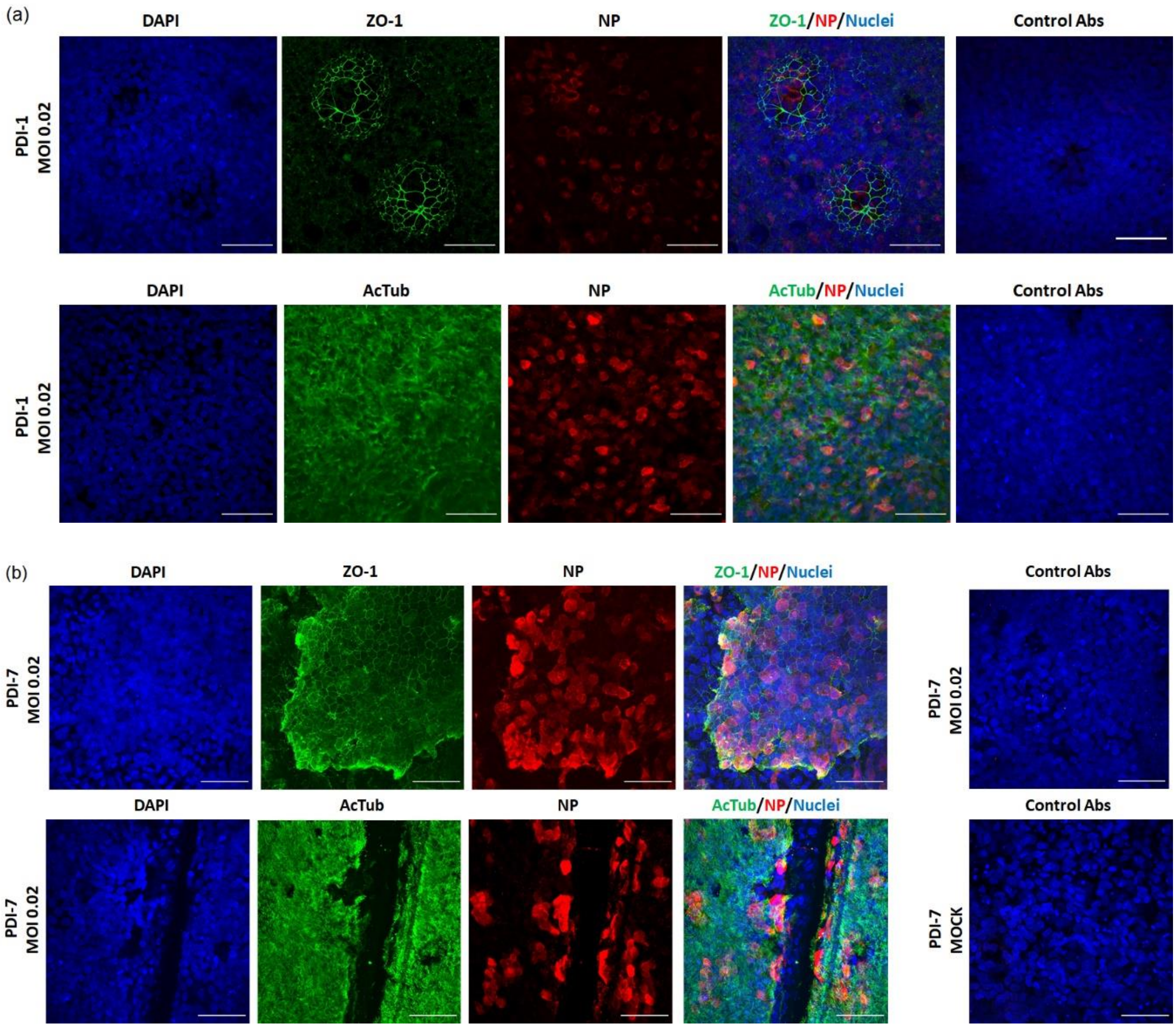

Figure S2. SARS-CoV-2 infected ALI-HNE. Adult (**a**) and child (**b**) ALI-HNE infected with VIC01. Immunofluorescent confocal microscopy staining for α-tubulin (AcTub, green) and nucleoprotein (NP, red) or control antibodies (Control Abs). Nuclei are blue (DAPI). Scale bar 50 µm. Extended data for Figure 1.

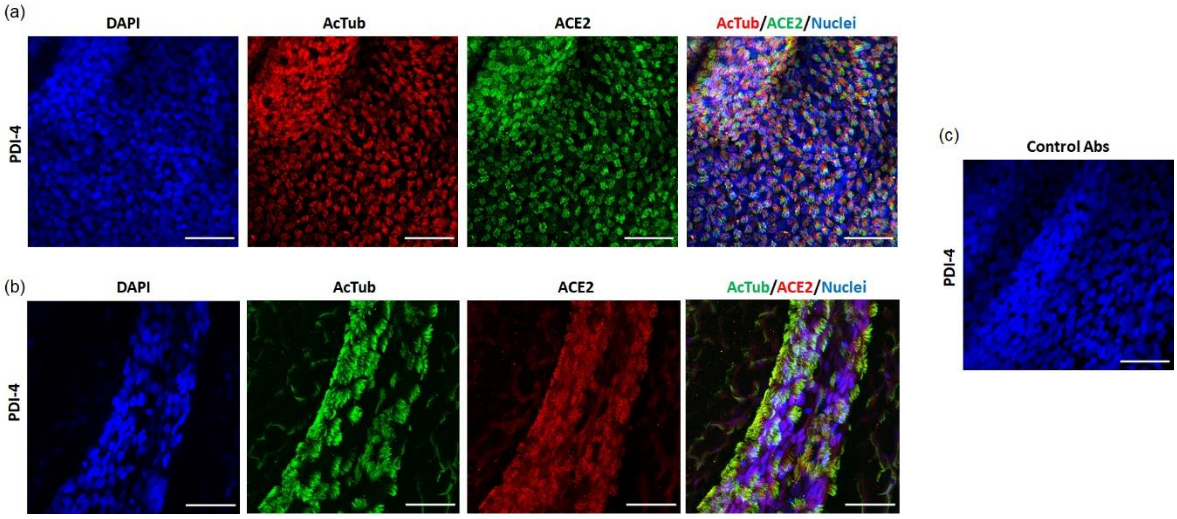

Figure S3. ACE2 is expressed by ALI-HNE. Immunofluorescent staining for ACE2 (green) and α-tubulin (red); nuclei are blue (DAPI) or control antibodies (Control Abs). Scale bar 50 µm. Extended data for Figure 2.

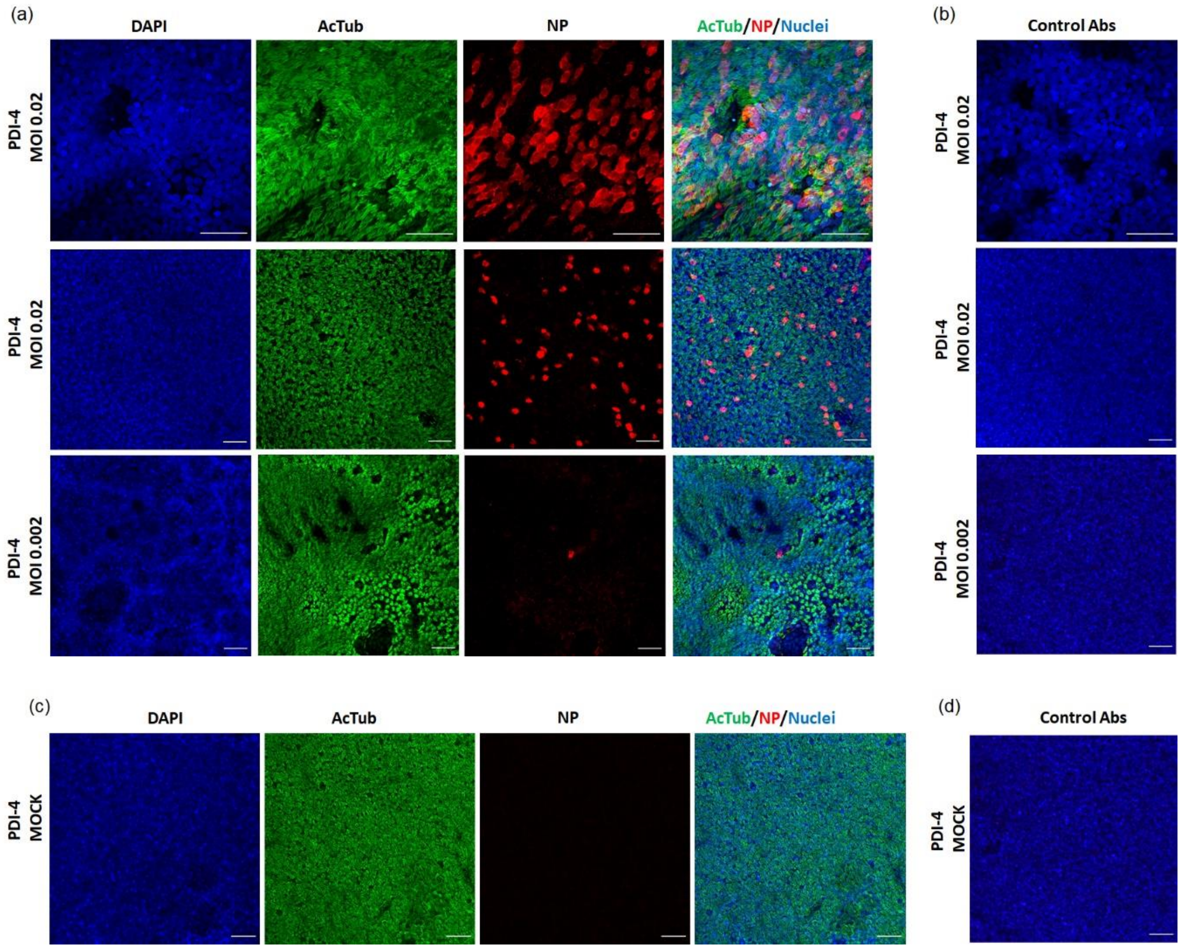

Figure S4. Dose dependent SARS-CoV-2 infection of PDI-4 ALI-HNE. ALI-HNE infected with VIC01 at MOI 0.02 and 0.002 (**a, b**) or mock (**b**). Immunofluorescent confocal microscopy staining for α-tubulin (AcTub, green) and nucleoprotein (NP, red) or control antibodies (Control Abs) are shown; nuclei are blue (DAPI). Scale bar 50 μm. Extended data for Figure 3.

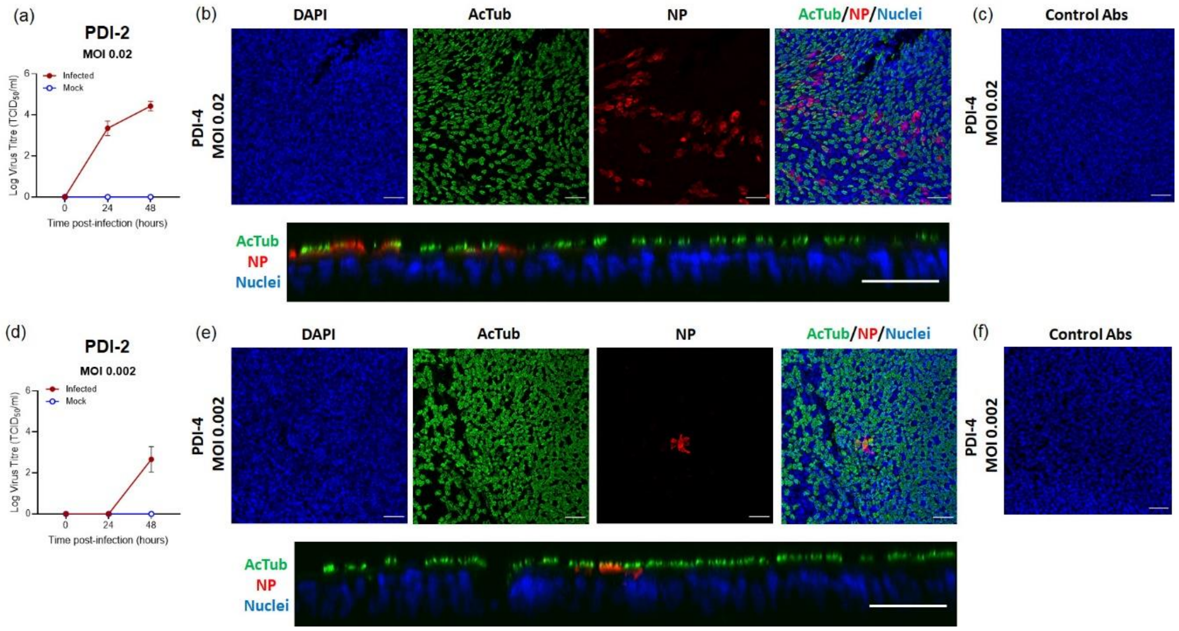

Figure S5. Dose dependent SARS-CoV-2 infection of PDI-2 ALI-HNE. ALI-HNE infected with VIC01 at MOI 0.02 (**a, b, c**) and 0.002 (**d**, **e**, **f**); (**a, d**) infectious virus (TCID50) in the apical wash harvested at the indicated times; (**b**, **c**, **e**, **f**) immunofluorescent confocal microscopy staining for α-tubulin (AcTub, green) and nucleoprotein (NP, red) or control antibodies (Control Abs); nuclei are blue (DAPI). Top and orthogonal views are shown. Scale bar 50 μm.

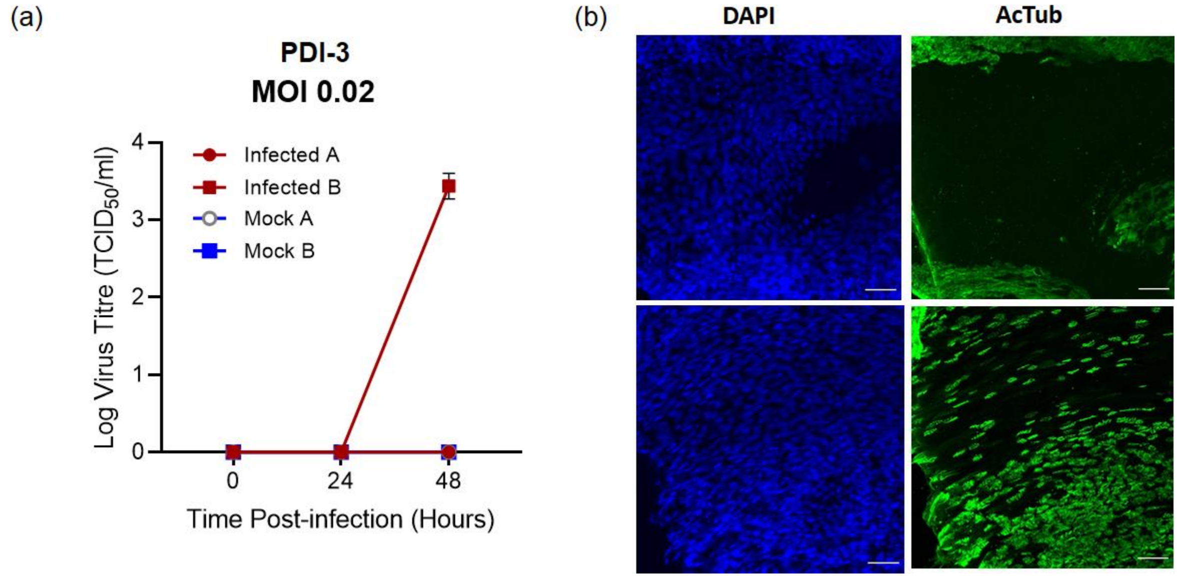

Figure S6. SARS-CoV-2 infected PDI-3 ALI-HNE. (**a**) Infectious virus (TCID50) in the apical wash harvested at the indicated times. (**b**) Immunofluorescent confocal microscopy staining for α-tubulin (AcTub, green) shows non-uniform ALI differentiation towards the ciliated cell type; nuclei are blue (DAPI). Scale bar 50 μm.

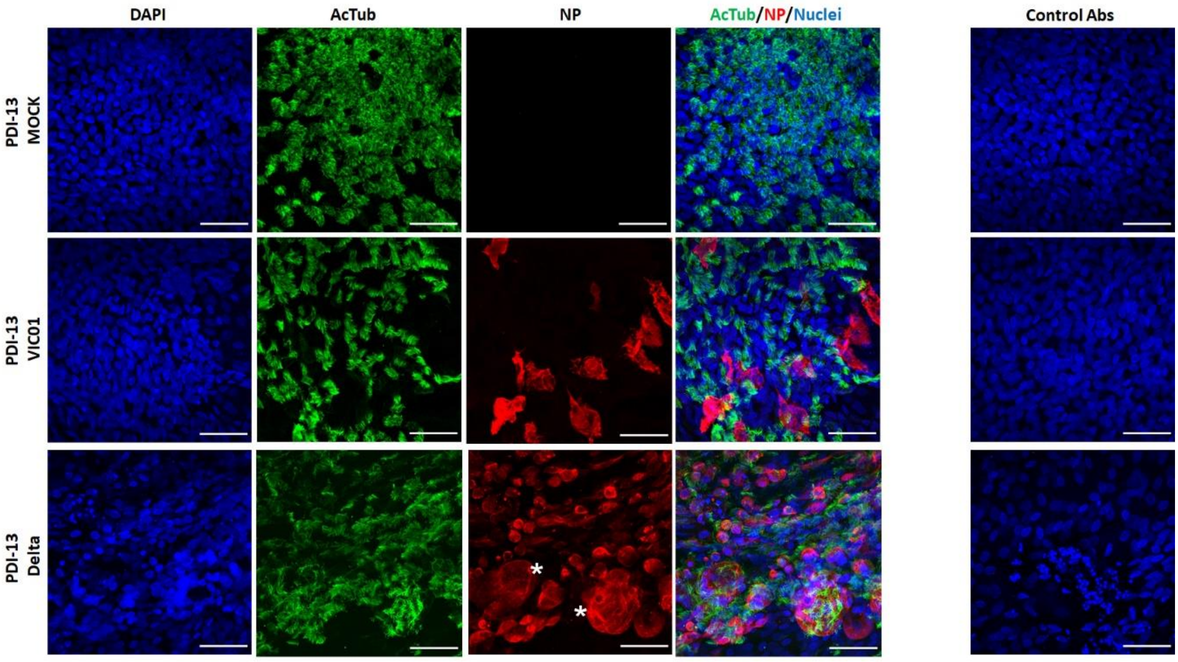

Figure S7. VIC01 and Delta SARS-CoV-2 infected ALI-HNE. Immunofluorescent confocal microscopy staining for α-tubulin (AcTub, green) and nucleoprotein (NP, red) or control antibodies (Control Abs); nuclei are blue (DAPI). *Indicates examples of syncytia formation. Scale bar 50 μm. Extended data for Figure 4.

Table S1. Antibodies used for immunofluorescent microscopy

Antibody	Supplier and catalogue number	Dilution
Acetylated α-tubulin	Sigma-Aldrich #T7451	1:250
Human ACE2	Sigma-Aldrich #HPA000288	1:100
ZO-1	Invitrogen # 33-9100	1:200
SARS Nucleocapsid Protein	Novus Biologicals #NB100-56683	1:200
Goat anti-Mouse Alexa Fluor 488	Invitrogen #A11001	1:450
Goat anti-Mouse Alexa Fluor 647	Invitrogen #A21236	1:450
Goat anti-Rabbit Alexa Fluor 488	Invitrogen #A1034	1:450
Goat anti-Rabbit Alexa Fluor 647	Invitrogen #A21244	1:450
